# Nutritional predictors for postoperative short-term and long-term outcomes of patients with gastric cancer

**DOI:** 10.1097/MD.0000000000003781

**Published:** 2016-06-17

**Authors:** Mitsuro Kanda, Akira Mizuno, Chie Tanaka, Daisuke Kobayashi, Michitaka Fujiwara, Naoki Iwata, Masamichi Hayashi, Suguru Yamada, Goro Nakayama, Tsutomu Fujii, Hiroyuki Sugimoto, Masahiko Koike, Hideki Takami, Yukiko Niwa, Kenta Murotani, Yasuhiro Kodera

**Affiliations:** a Department of Gastroenterological Surgery (Surgery II), Nagoya University Graduate School of Medicine, Nagoya; b Center for Clinical Research, Aichi Medical University, Nagakute, Japan.

**Keywords:** adjuvant chemotherapy, gastric cancer, postoperative complication, prognosis, prognostic nutrition index

## Abstract

Evidence indicates that impaired immunocompetence and nutritional status adversely affect short-term and long-term outcomes of patients with cancer. We aimed to evaluate the clinical significance of preoperative immunocompetence and nutritional status according to Onodera's prognostic nutrition index (PNI) among patients who underwent curative gastrectomy for gastric cancer (GC).

This study included 260 patients with stage II/III GC who underwent R0 resection. The predictive values of preoperative nutritional status for postoperative outcome (morbidity and prognosis) were evaluated. Onodera's PNI was calculated as follows: 10 × serum albumin (g/dL) + 0.005 × lymphocyte count (per mm^3^).

The mean preoperative PNI was 47.8. The area under the curve for predicting complications was greater for PNI compared with the serum albumin concentration or lymphocyte count. Multivariate analysis identified preoperative PNI < 47 as an independent predictor of postoperative morbidity. Moreover, patients in the PNI < 47 group experienced significantly shorter overall and disease-free survival compared with those in the PNI ≥ 47 group, notably because of a higher prevalence of hematogenous metastasis as the initial recurrence. Subgroup analysis according to disease stage and postoperative adjuvant treatment revealed that the prognostic significance of PNI was more apparent in patients with stage II GC and in those who received adjuvant chemotherapy.

Preoperative PNI is easy and inexpensive to determine, and our findings indicate that PNI served as a significant predictor of postoperative morbidity, prognosis, and recurrence patterns of patients with stage II/III GC.

## 1 Introduction

Gastric cancer (GC) is the 2nd most frequent cause of cancer deaths worldwide.^[1,2]^ Patients with stage II/III GC sometimes suffer from recurrence even after curative gastrectomy,^[3–5]^ which is a complex procedure associated with relatively high morbidity. Thus identifying predictive factors for surgical morbidity as well as disease recurrence and long-term survival will enhance efforts to provide patients with individualized perioperative management.

Impaired nutritional status leads to increased susceptibility to infection, protracted wound healing, impaired blood clotting and vessel-wall fragility, increased frequency of postoperative complications, and accelerated tumor progression through the suppression of tumor immunity.^[6,7]^ Evaluation of preoperative immunocompetence and nutritional status can be useful in the search for a strategy to improve short-term and long-term outcomes of patients with cancer. Since the concept of a prognostic nutrition index (PNI) was 1st suggested in 1980, various PNIs were considered as integral parameters that can reflect immunocompetence and nutritional status with greater accuracy compared with that of a single variable.^[8–10]^ Onodera's PNI (10 × albumin g/dL) + (0.005 × lymphocyte count/mm^3^) is associated with outcomes of patients with different malignancies.^[9,11–13]^ Advanced GC causes debilitating malnutrition and deterioration of the immune response.^[3]^ Further, gastrointestinal obstruction and bleeding can further impair a patient's immunocompetence and nutritional status.^[4]^ Thus it is reasonable to conclude that Onodera's PNI is predictive of adverse outcomes; however, the clinical significance of the PNI remains controversial when applied to patients with GC.

The aim of the present study was to evaluate the influence of preoperative immunocompetence and nutritional status with particular focus on using Onodera's PNI to predict short-term and long-term outcomes of patients with stage II/III GC.

## 2 Materials and methods

### 2.1 Ethics

This study conformed to the ethical guidelines of the World Medical Association's Declaration of Helsinki–Ethical Principles for Medical Research Involving Human Subjects. Patients granted written informed consent for surgery and the use of clinical data, which was approved by the Institutional Review Board.

### 2.2 Patients

We reviewed the records of 1193 patients with GC who underwent surgery at the Department of Gastroenterological Surgery, Nagoya University between January 1999 and August 2015. We selected 260 patients for this study who met the inclusion criteria as follows: no preoperative treatment, stage II/III GC according to the 7th Edition of the TNM Classification of Malignant Tumors,^[14]^ integrity of data, pathological evaluation of ≥15 lymph nodes, and no preoperative administration of blood-products.

### 2.3 Treatment

Patients underwent D2 gastrectomy, and the surgeon selected the reconstruction method. Since 2007, adjuvant chemotherapy with S-1 (an oral fluoropyrimidine derivative) was available as a standard option for treating patients who were eligible for the current study, unless contraindicated by a patient's condition.^[15,16]^ The chemotherapy protocol implemented after recurrence was determined by the attending physicians according to the available evidence, the patient's physical condition, and with the patient's consent. Postoperative follow-up, which was conducted according to the Japanese Gastric Cancer Treatment Guidelines, included physical examination, laboratory tests, and enhanced computed tomography (chest and abdominal cavity) once every 6 months for 5 years or until death.^[17,18]^

### 2.4 Investigational variables

The variables investigated as candidate immune-nutritional factors, which can be rapidly measured in every hospital, were as follows: body mass index (BMI), total lymphocyte count (TLC), hemoglobin concentration, platelet count, total protein, albumin, cholesterol, cholinesterase, urea nitrogen, neutrophil–lymphocyte ratio (NLR), platelet–lymphocyte ratio (PLR), and Onodera's PNI (PNI = 10 × albumin g/dL + 0.005 × lymphocyte count/mm^3^).^[12]^ Nutritional factors were measured within 3 days before surgery. Oral nutritional support using various products for enteral alimentation to enhance the immune-nutritional status was not administered to patients during the study period. Postoperative factors evaluated using the Clavien–Dindo classification system were as follows: grades III (complications requiring surgical, endoscopic, or radiological intervention), IV (life-threatening complications requiring intensive care), and V (death).^[19]^

### 2.5 Statistical analysis

We performed receiver operating characteristic curve analysis of postoperative complications to evaluate the ability of the optimal cut-off value of the preoperative PNI of interest to predict postoperative outcomes. Goodness of fit was assessed by calculating the area under the curve (AUC), and the optimal cut-off value was determined using the Youden index.^[20]^ The qualitative χ^2^ and quantitative Mann–Whitney tests were used to compare the 2 groups. Potential risk factors for postoperative complications were evaluated using binomial logistic analyses. Survival rates were estimated using the Kaplan–Meier method, and the overall differences between curves were compared using the log-rank test. The univariate Cox proportional hazards model was used to evaluate the hazard ratio for overall survival relative to each variable.^[21,22]^ Variables with *P* < 0.05 were included in the multivariate analysis to identify independent factors. Statistical analysis was performed using JMP 10 software (SAS Institute Inc., Cary, NC). *P* < 0.05 represents a statistically significant difference.

## 3 Results

### 3.1 Patients’ characteristics

The demographics and oncological characteristics of the 260 patients enrolled are shown in Table 1. Patients were diagnosed with stages IIA (n = 69), IIB (n = 59), IIIA (n = 38), IIIB (n = 52), and IIIC (n = 42). Total gastrectomy was performed to treat 104 patients (40%). The mean preoperative PNI was 47.8 ± 6.4 (standard deviation).

**Table 1:**
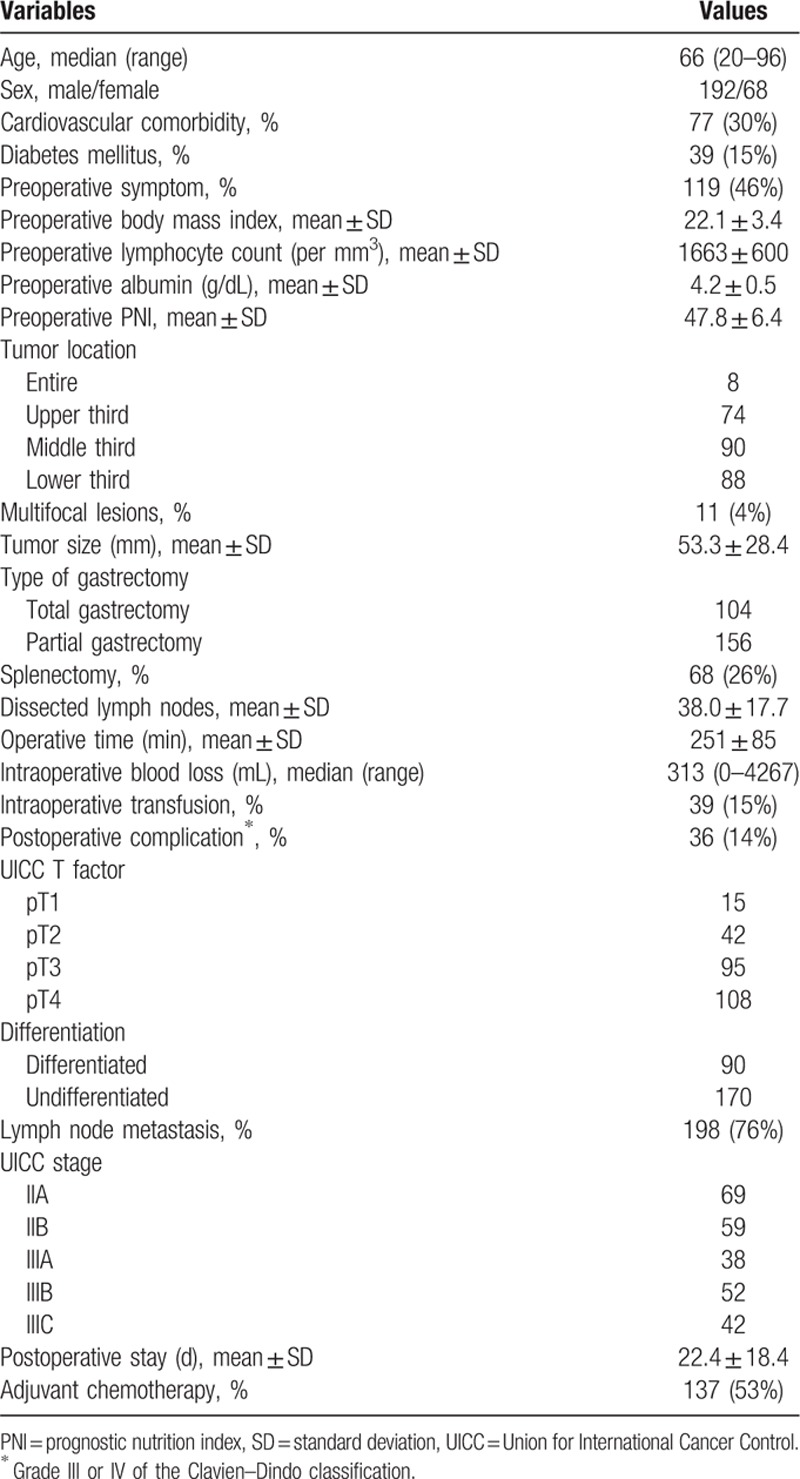
Demographics and perioperative clinical characteristics of 260 patients.

### 3.2 Preoperative nutritional status and postoperative complications

**Figure 1:**
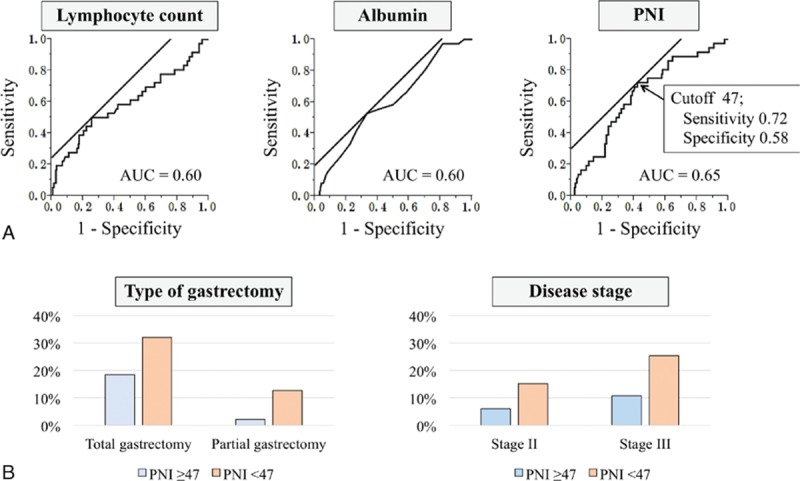
(A) Receiver operating characteristic curves for lymphocyte counts, serum albumin, and PNI as predictive factors for postoperative complications. The value of the area under the curve (AUC) was highest for PNI. (B) Incidence of postoperative complications according gastrectomy type and disease stage. PNI = prognostic nutrition index.

Thirty-six patients (14%) had grade III or higher postoperative complications, including anastomosis leakage (n = 17, 7%), leakage of pancreatic fluids (n = 9, 3%), and intra-abdominal abscess (n = 5, 2%). The AUC values indicating the predictive power of the postoperative complications were as follows: BMI, 0.54; TLC, 0.60; hemoglobin, 0.59; platelet count, 0.54; total protein, 0.55; albumin, 0.60; cholesterol, 0.57; cholinesterase, 0.56; urea nitrogen, 0.57; NLR, 0.62; and PLR, 0.63. Onodera's PNI was highest at AUC = 0.65, and the optimal cut-off value for predicting complications was 47 (sensitivity = 72%, specificity = 58%) (Fig. 1A).

Compared with patients with preoperative PNI ≥ 47, those with PNI < 47 were significantly older, had lower preoperative BMIs and larger macroscopic tumor sizes, and were more often administered intraoperative transfusions (Table 2). Further, patients with preoperative PNI < 47 required longer hospitalization after surgery. In contrast, there was no significant difference between patients with PNI values above or below the cutoff associated with the comorbidity, tumor location, and disease stage (Table 2).

**Table 2:**
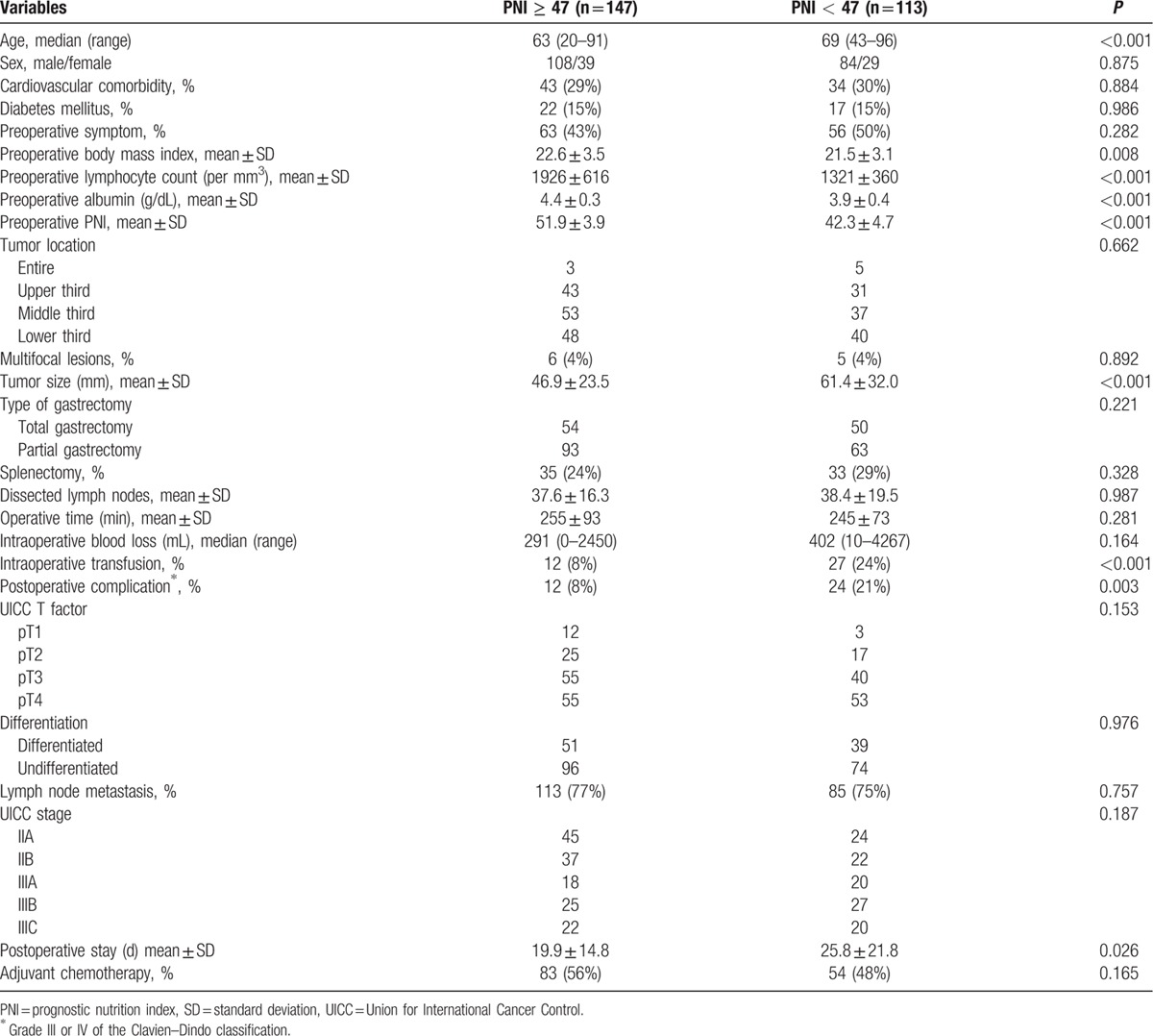
Comparison of characteristics between the PNI ≥ 47 and PNI < 47 groups.

Logistic regression analyses were performed to identify factors associated with postoperative complications. Multivariate analysis incorporating these factors as covariates identified preoperative PNI < 47 as an independent predictor of complications (odds ratio, 2.41; 95% confidence interval [CI], 1.03–5.84; *P* = 0.042) as well as operative time ≥ 240 min and intraoperative transfusion (Table 3). Subgroup analyses conducted according to the type of gastrectomy and disease stage revealed that subgroups with PNI < 47 experienced a higher incidence of postoperative complications (Fig. 1B).

**Table 3:**
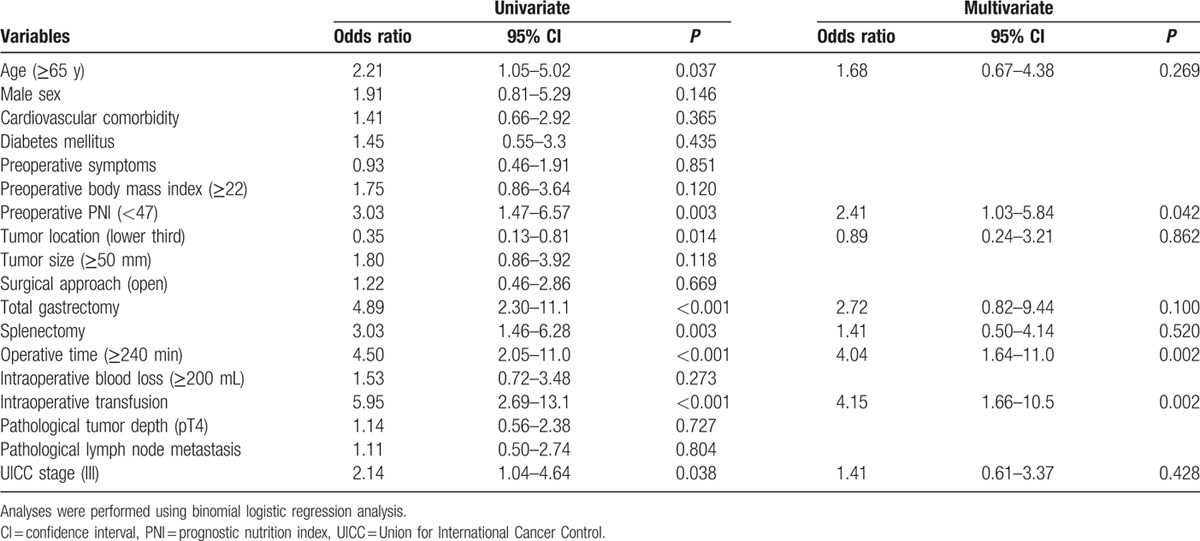
Univariate and multivariate analyses of risk factors for postoperative complications.

### 3.3 Prognostic impact of preoperative PNI

**Figure 2:**
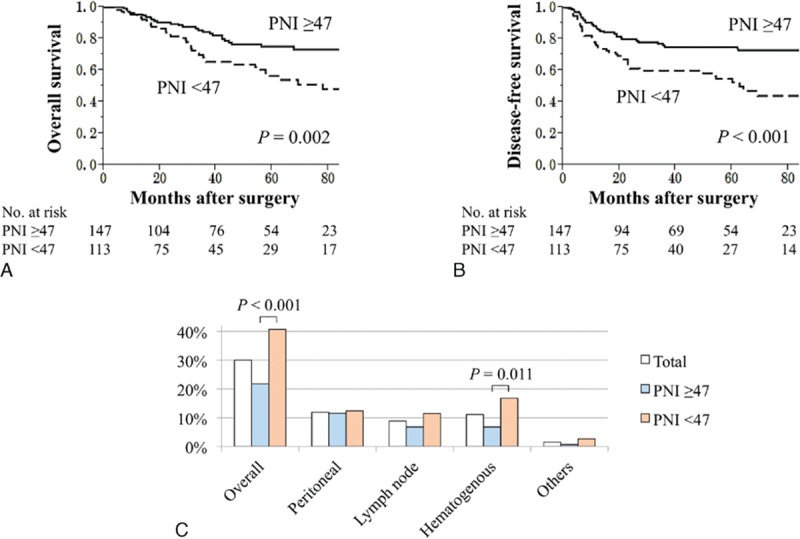
Survival analyses and recurrence patterns of 260 patients with stage II/III gastric cancer. The PNI <47 group was more likely to have (A) shorter overall and (B) disease-free survival compared with the PNI ≥ 47 group. (C) Prevalence of the site of initial recurrence. PNI = prognostic nutrition index.

The overall survival of patients in the PNI < 47 group was significantly shorter after curative gastrectomy (5-year survival rates: PNI < 47, 56%; PNI ≥ 47, 75%; *P* = 0.002) (Fig. 2A). The PNI < 47 group experienced significantly shorter disease-free survival (3-year survival rates: PNI < 47, 60%; PNI ≥ 47, 76%; *P* < 0.001) (Fig. 2B). The overall recurrence rate of the PNI < 47 group was significantly higher (PNI < 47, 41%; PNI ≥ 47, 22%; *P* < 0.001). Interestingly, the PNI < 47 group experienced a significantly higher prevalence of hematogenous metastasis (liver, lung, and bone) as initial recurrence (PNI < 47, 17%; PNI ≥ 47, 7%; *P* = 0.011), whereas both groups experienced equivalent frequencies of peritoneal recurrence (Fig. 2C).

### 3.4 Subgroup analyses

**Figure 3:**
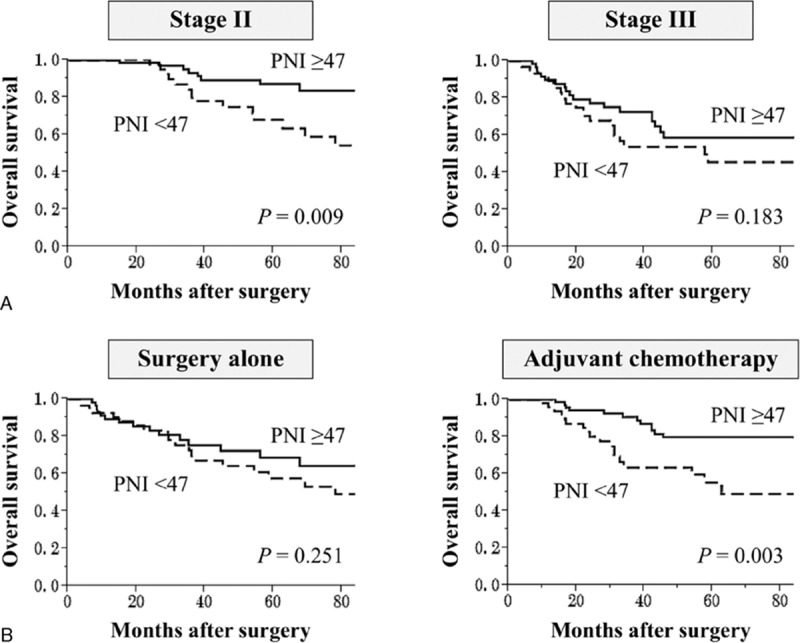
Subgroup analyses of the prognostic impact of PNI according to (A) disease stage and (B) administration of adjuvant chemotherapy. Survival curves indicate the overall survival rate. PNI = prognostic nutrition index.

To explore further the clinical implications of PNI for the long-term outcome of patients with stage II/III GC, subgroup analyses were conducted according to disease stage and administration of adjuvant chemotherapy. The difference between the survival curves of the PNI ≥ 47 and PNI < 47 groups was statistically significant for patients with stage II GC (Fig. 3A). Multivariate analysis identified PNI < 47 as an independent prognostic factor for mortality (hazard ratio, 3.04; 95% CI, 1.30–7.63; *P* = 0.010) for patients with stage II GC (Table 4). Differences in certain characteristics of the 2 groups were revealed that when patients were subdivided according to the administration of adjuvant chemotherapy. For patients who received adjuvant chemotherapy following curative gastrectomy, there was a significant difference between the PNI < 47 and PNI < 47 groups (5-year survival rates: PNI < 47, 44%; PNI ≥ 47, 68%; *P* = 0.003). In contrast, the difference in survival was not significant for patients who underwent surgery alone (Fig. 3B).

**Table 4:**
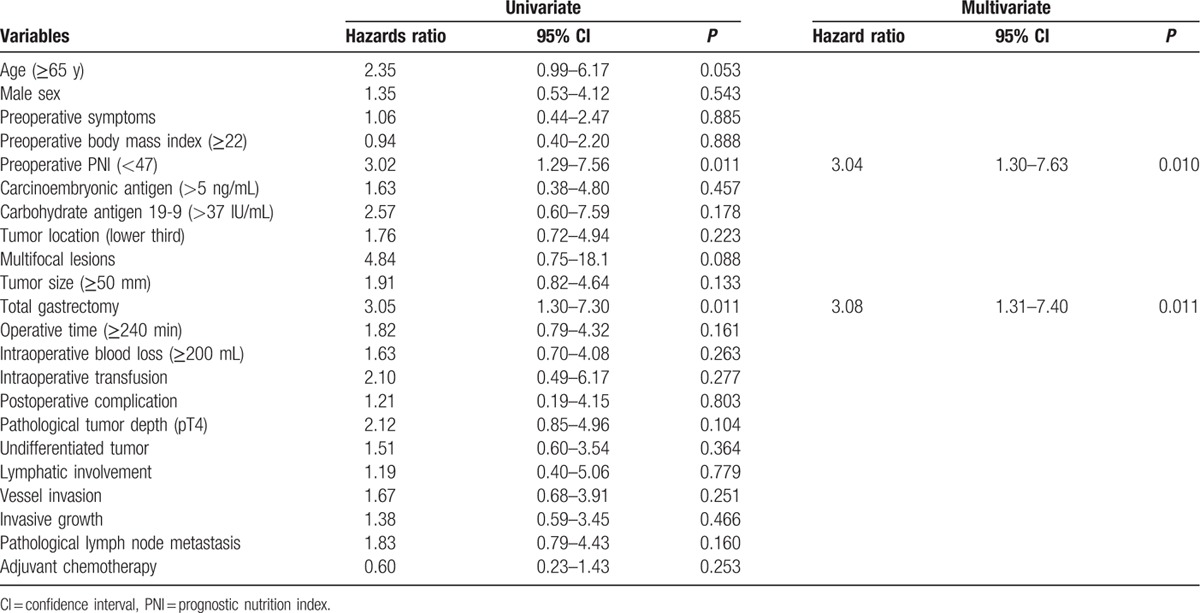
Prognostic factors for overall survival of 128 patients with stage II gastric cancer.

## 4 Discussion

Patients with GC are frequently malnourished.^[4]^ In addition to the effects of poor oral nutritional intake and protein loss from the primary lesion, cancer cells secrete cytokines such as tumor necrosis factor-alpha that adversely affect catabolic metabolism.^[23,24]^ Moreover, nutritional status and immunocompetence are closely related.^[12,25]^ Protein and energy malnutrition can cause not only changes in physical appearance but typically reduce body weight, muscle mass, serum albumin levels, TLC, the number of helper T cells, interleukin (IL)-2 and IL-3 levels, and T-cell blastogenic responses.^[26]^ The cumulative consequences of these alterations adversely affect short-term and long-term outcomes through increased postoperative complications attributed to tissue vulnerability, impaired wound healing, susceptibility to infection, and accelerated tumor progression caused by compromised tumor immunity.^[6,7,9]^ Moreover, inflammatory immune responses involving the gastric mucosa, such as chronic gastritis following *Helicobacter pylori* infection, play a prominent role in the pathogenesis of GC.^[27,28]^ Therefore, evaluating a patient's immunocompetence is an important consideration in the management of GC.

In the present study, we show that PNI was the best predictor of the incidence of postoperative complications. The use of an integrated index was justified, because the AUC value of PNI was greater compared with those of the PNI components albumin level and lymphocyte count. Moreover, patients with low preoperative PNI more frequently experienced complications independent of the type of gastrectomy and disease stage. To translate our findings to the clinic, perioperative nutritional intervention will be required. Preoperative enteral alimentation for malnourished surgical patients with cancers of the digestive system improves postoperative outcomes through significant elevations of albumin levels and lymphocyte counts.^[29–31]^

The European Society for Parenteral and Enteral Nutrition Guidelines for adult parenteral nutrition (2009) and the Enhanced Recovery after Surgery Society (2012) recommend implementing assessment and management of nutrition, mainly through enteral alimentation for patients with poor oral intake and those undergoing major surgery.^[32–34]^ Therefore, the PNI may be useful for assessment of preoperative nutritional status and the efficacy of nutritional support.

Moreover, we show here that preoperative PNI was significantly associated with long-term outcomes (disease-free and overall survival) of patients with stage II/III GC, which is consistent with previous studies of different malignancies.^[9,10,35]^ Further, we linked decreased PNI to increased risk of hematogenous metastasis but not peritoneal dissemination. A reasonable explanation for these findings is that immunocompetence and nutritional status are more relevant to hematogenous metastasis of circulating tumor cells because of the effects of compromised tumor immunity associated with the types and levels of circulating cytokines. In the clinic, PNI may therefore serve to identify patients at risk of poor prognosis as well predicting the sites of disease recurrence. In particular, intensive postoperative surveillance for hematogenous metastasis, for example, Gd-EOB-DTPA enhanced magnetic resonance imaging or contrast-enhanced ultrasound of the liver may be advisable for patients with low preoperative PNI.^[36]^

An important finding to be emphasized is that PNI was a paramount prognostic factor for patients with Stage II GC. Although tumor depth and nodal status were not. The requirement for adjuvant chemotherapy to treat patients with stage II GC with the same intensity as stage III is now under discussion worldwide, because patients with stage II GC are expected to have a favorable prognosis compared with those with stage III GC.^[15,16,37,38]^ According to the present results, preoperative PNI may serve as an effective indicator for selecting patients with stage II GC who are eligible for intense nutritional intervention.

We found it interesting that patients with low preoperative PNI derived no significant benefit from adjuvant chemotherapy. To account for this observation, we suggest that a reduced therapeutic response to adjuvant chemotherapy of patients with impaired immune-nutritional status reflected reduced tolerance to chemotherapy, which caused further deterioration of immunocompetence induced by the adverse effects of chemotherapy that accelerated tumor progression. Although an evaluation of the correlation of preoperative PNI with time-to-treatment failure and relative dose intensity of adjuvant chemotherapy must be conducted in the future, we recommend that clinicians consider implementing regimens for adjuvant therapy and nutritional supportive care according to preoperative PNI.

The present study is limited because of its retrospective design and the limited number of subjects, which may have biased the data. Data on preoperative body constitution and muscle mass may contribute to the discussion of the relevance between PNI and sarcopenia. Further, insufficient immune-nutritional data, such as decreased cytokine levels, may have prevented us from acquiring a better understanding of the underlying mechanism of tumor progression caused by immunosuppression. At the moment, our data cannot be extrapolated to populations other than Asians. Prospective studies of perioperative nutritional support according to preoperative PNI are therefore required to pursue the clinical significance of PNI in GC.

In conclusion, our results indicate that preoperative PNI was associated with postoperative morbidity, prognosis, and recurrence patterns of patients with stage II/III GC after curative gastrectomy. Evaluation of preoperative immunocompetence and nutritional status may facilitate the design of more effective perioperative management strategies, guide long-term follow-up studies, and illuminate options for adjuvant chemotherapy.
